# Are obligatory apomicts invested in the pollen tube transmitting tissue? Comparison of the micropyle ultrastructure between sexual and apomictic dandelions (Asteraceae, Lactuceae)

**DOI:** 10.1007/s00709-015-0765-x

**Published:** 2015-02-05

**Authors:** Bartosz J. Płachno, Piotr Świątek, Małgorzata Kozieradzka-Kiszkurno, Ľuboš Majeský, Jolanta Marciniuk, Piotr Stolarczyk

**Affiliations:** 1 Department of Plant Cytology and Embryology, Jagiellonian University in Kraków, Kraków, 9 Gronostajowa St., 30-387 Kraków, Poland; 2 Department of Animal Histology and Embryology, University of Silesia, 9 Bankowa St., 40-007 Katowice, Poland; 3 Department of Plant Cytology and Embryology, University of Gdańsk, 59 Wita Stwosza St., 80-308 Gdańsk, Poland; 4 Department of Botany, Faculty of Science, Palacký University, 11 Šlechtitelů St., 783 71 Olomouc, Czech Republic; 5 Department of Botany, Siedlce University of Natural Sciences and Humanities, 12 Prusa St., 08-110 Siedlce, Poland; 6 Unit of Botany and Plant Physiology, Institute of Plant Biology and Biotechnology, Faculty of Horticulture, University of Agriculture in Kraków, 54 Al. 29 Listopada, 31-425 Kraków, Poland

**Keywords:** Ovule, Integument, Transmitting tissue, Gynoecium, Dandelions, Asteraceae, Apomixis

## Abstract

With the exception of the sunflower, little information concerning the micropyle ultrastructure of the family Asteraceae is available. The aim of our study was to compare the micropyle structure in amphimictic and apomictic dandelions. Ultrastructural studies using buds and flowers during anthesis have been done on the micropyle of the sexual and apomictic *Taraxacum*. In all of the species that were examined, the micropylar canal was completely filled with ovule transmitting tissue and the matrix that was produced by these cells. The ovule transmitting tissue was connected to the ovarian transmitting tissue. The micropyle was asymmetrical because the integument epidermis that forms the transmitting tissue was only on the funicular side. There was a cuticle between the obturator cells and epidermal cells on the other side of integument. The micropylar transmitting tissue cells and theirs matrix reached the synergid apex. The cytoplasm of the transmitting tissue cells was especially rich in rough endoplasmic reticulum (ER), dictyosomes, and mitochondria. No major differences were detected between the micropyle structure of the amphimictic and apomictic species; thus, a structural reduction of obturator does not exist. The ovule transmitting tissue is still active in apomictic dandelions despite the presence of the embryo and endosperm. Differences and similarities between the micropyle structure in the Asteraceae that have been studied to date are discussed.

## Introduction

The Asteraceae family in which there are 12 subfamilies according to Funk et al. (2009) is one of the largest Angiosperm families. Surprisingly, to date, only a few studies have described the micropyle structure in only a few members of this family—in the barnadesioid *Arnaldoa macbrideana* (Erbar and Leins 2000), the asteroids *Buphthalmum salicifolium* (Erbar 2003) and *Helianthus annuus* (Yan et al. 1991), and the cichorioids *Cichorium intybus* (Erbar and Enghofer 2001) and *Chondrilla juncea* (Kościńska-Pająk et al 2005; Kościńska-Pająk 2006). According to Erbar (2003 and references therein), the ovarian transmitting tissue in *A. macbrideana* (a member of Barnadesioideae, which has a basal position in Asteraceae) comes into close contact with the transmitting tissue on the long funiculus. The funicular transmitting tissue consists of two lines which are fused beneath the entrance of the micropyle and later enter the micropyle. In more advanced members of Asteraceae, the funiculus is shorter; however, a similar course of the transmitting tissue has been described (Erbar 2003).

The ultrastructure of the micropylar transmitting tissue has only been examined in two species of Asteraceae—the asteroid *H. annuus* (Yan et al. 1991) and the cichorioid *C. juncea* (Kościńska-Pająk et al. 2005; Kościńska-Pająk 2006). Yan et al. (1991) showed that the micropyle in the sunflower was asymmetrical. The cells were on the funicular side forming transmitting tissue. The cells that were distal to the funicle were rich in rough endoplasmic reticulum (ER) and lipid bodies but lacked large intercellular spaces. According to Kościńska-Pająk (2006), a part of the funiculus tissue protruded toward the ovule micropyle and formed a micropylar transmitting tissue in the obligatory apomictic *C. juncea*. Moreover, at the stage of embryo sac maturation, the space of the micropylar canal was filled with an extracellular matrix substance that was produced during the secretory activity and lysis of the transmitting tissue cells. The ultrastructure of the transmitting tissue was only shown by Kościńska-Pająk (2006) on Tab XXXII 2; however, there was no description of the cell details.

The giant *Taraxacum* Wigg. genus., which comprises about 3,000 species as well as some new species, is still being described, e.g., *Taraxacum pomposum* Štěpánek & Kirschner (Štěpánek and Kirschner 2013), *Taraxacum riparium* Štěpánek, Kirschner, Kirchmeier & Meierott (Štěpánek et al. 2013), *Taraxacum palmeri* Walter Scott & T.C.G. Rich (Scott and Rich 2013), and *Taraxacum zajacii* J. & P. Marciniuk (Marciniuk et al. 2012). There is a correlation between the ploidy level and the mode of reproduction in this genus because diploid, and very rarely, also tetraploid (Kirschner and Štěpánek 1998) species are amphimictic, whereas polyploids are obligate apomicts (Richards 1973; den Nijs and Menken 1996; den Nijs 1997; Hörandl 2010). Apomixis within the genus *Taraxacum* is a type of meiotic diplospory, and the development of both the embryo and the endosperm does not require double fertilization. The development of the progeny is fully independent of the male gametophyte (Asker and Jerling 1992). Recently, *Hieracium* (e.g., Tucker et al. 2012; Okada et al. 2013; Ogawa et al. 2013) and *Taraxacum* have been the most important models for studies of apomictic reproduction in the Asteraceae family (e.g., Van der Hulst et al. 2000, 2003; Van Dijk 2003; Majeský et al. 2012). We recently studied the ovule anatomy and egg apparatus structure in sexual and apomictic dandelions (Musiał et al. 2013; Płachno et al. 2014a). Since both endosperm and embryo development are independent of pollination, it seemed desirable to check whether this is reflected in the micropyle/ovule transmitting tissue structure.

We consider our study to be a preliminary step for a future study of micropyle activity and pollen tube growth in the apomictic *Taraxacum* species. However, some basic knowledge about the micropyle in sexual and apomictic *Taraxacum* species is strongly needed before any analyses of apomicts. We would like to test the hypothesis that there is a structural reduction of the micropylar transmitting tissue in apomictic dandelions in comparison with their sexual counterparts. Since embryo and endosperm development does not require fertilization, a reduction of the transmitting tissue can be expected. The second aim is to check whether there are any major differences between the micropyle structures in the members of different *Taraxacum* sections (*Palustria* versus *Taraxacum*).

Our paper not only presents the preliminary studies of the micropyle structure in the genus *Taraxacum* but also shows that even the ovule of apomicts that has an endosperm and embryo has vital transmitting tissue cells; thus, there is no barrier for the pollen tube to reach the ovule. Therefore, apomictic dandelions did not reduce some of the structural and developmental characters that are not effectively used by apomictic individuals.

## Materials and methods

### Plant material

We studied and compared the micropyle structure among several sexual and apomictic *Taraxacum* species. Sexual species were represented by two diploid species—*Taraxacum tenuifolium* (Hoppe & Hornsch.) Koch (*T.* sect*. Palustria*) and *Taraxacum linearisquameum* Soest [*T*. sect. *Taraxacum*; syn *T.* sect. *Ruderalia* (Kirschner and Štěpánek 2011)]. The apomictic sample was represented by various apomictic taxa from the *T*. sect. *Taraxacum* (*Taraxacum officinale* agg., from Palacký University, Olomouc, Czech Republic and specimens that were collected in Kraków-Podgórze, Poland). Studies were carried out on flowers before and during anthesis.

Vouchers of the taxa that were examined were deposited in the herbarium of Jagiellonian University in Kraków (KRA), with the exception of *T. officinale s.l.* (clone SA-B), which was deposited in the herbarium of Department of Botany, Palacký University.

### Light and electron microscopy studies

The preparation of the samples for transmission electron microscopy (TEM) followed the procedure described by Płachno and Świątek (2009) and Płachno (2011). Briefly, ovaries were fixed in 2.5 % formaldehyde and 2.5 % glutaraldehyde in a 0.05 M cacodylate buffer (pH 7.0) for 2 days for the electron microscopy studies. The material was postfixed in 1 % OsO_4_ in a cacodylate buffer for 24 h at ~4 °C, rinsed in the same buffer, treated with 1 % uranyl acetate in distilled water for 1 h, dehydrated with acetone, and embedded in an Epoxy Embedding Medium Kit (Fluka) or in Spurr's resin. Semithin sections were stained with methylene blue and examined using an Olympus BX60 microscope. The periodic acid-Schiff (PAS) reaction was used to visualize the total carbohydrates of insoluble polysaccharides (Wędzony 1996). Additionally, the material that had been embedded in Technovit 7100 (Kulzer, Germany; for procedure, see Płachno et al. 2014b; Kolczyk et al. 2014) was also used for the PAS reaction. All of the results were the same—the total carbohydrates of insoluble polysaccharides stained magenta to purplish red.

Ultrathin sections were cut on a Leica ultracut UCT ultramicrotome. After contrasting with uranyl acetate and lead citrate, the sections were examined using a Hitachi H500 electron microscope at 75 kV.

## Results

### Micropyle ultrastructure in sexual species

**Fig. 1:**
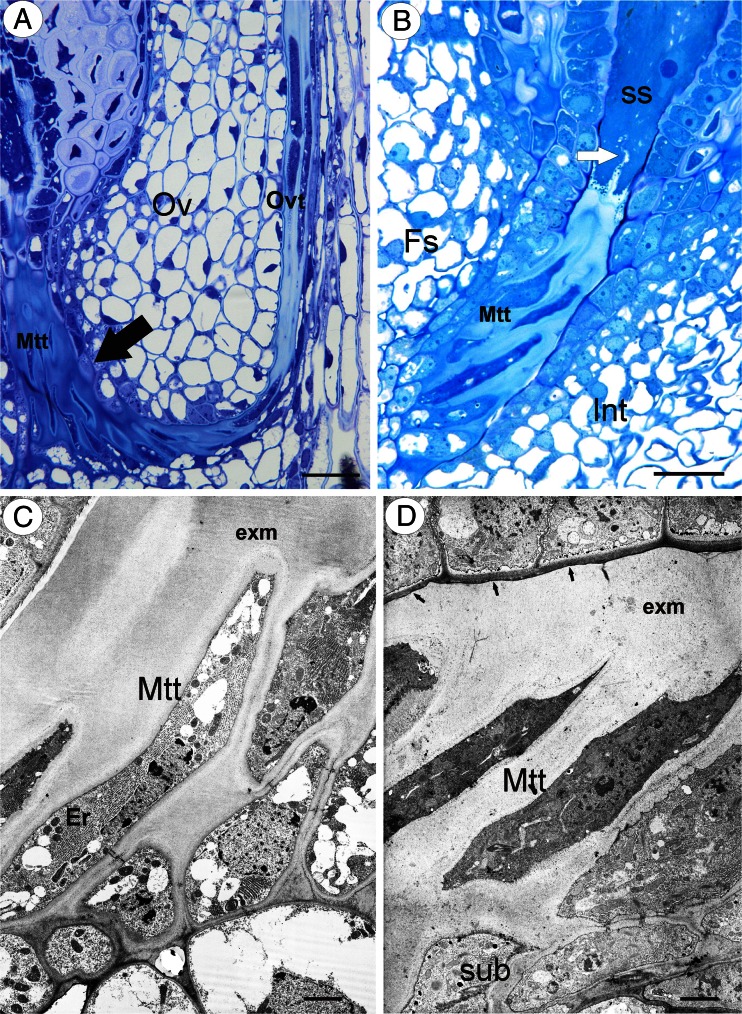
Transmitting tissue structure in sexual dandelions. **a** *T. linearisquameum* semithin (longitudinal) section through an ovule (*Ov*) and part of an ovary showing the transmitting tissue: micropylar transmitting tissue (*Mtt*, *black arrow*) and ovary transmitting tissue (*Ovt*), *bar* = 20 μm. **b** *T. tenuifolium* semithin section through the micropylar part of an ovule; integument (*Int*), micropylar transmitting tissue (*Mtt*), funicular side (*Fs*), synergids (*ss*), and filiform apparatus (*white arrow*). *Bar* = 20 μm. **c** *T. linearisquameum* Electron micrograph showing micropylar transmitting tissue cells (*Mtt*); extracellular matrix (*exm*) and endoplasmic reticulum (*Er*). *Bar* = 1.1 μm. **d** *T. tenuifolium* Electron micrograph showing micropylar transmitting tissue cells (*Mtt*); extracellular matrix (*exm*) and subepidermal cells (*sub*). *Bar* = 2.5 μm

The ovule transmitting tissue was connected to the ovarian transmitting tissue (Fig. 1a), which runs in the region of the two reduced septal ledges (Fig. 1a).

**Fig. 2:**
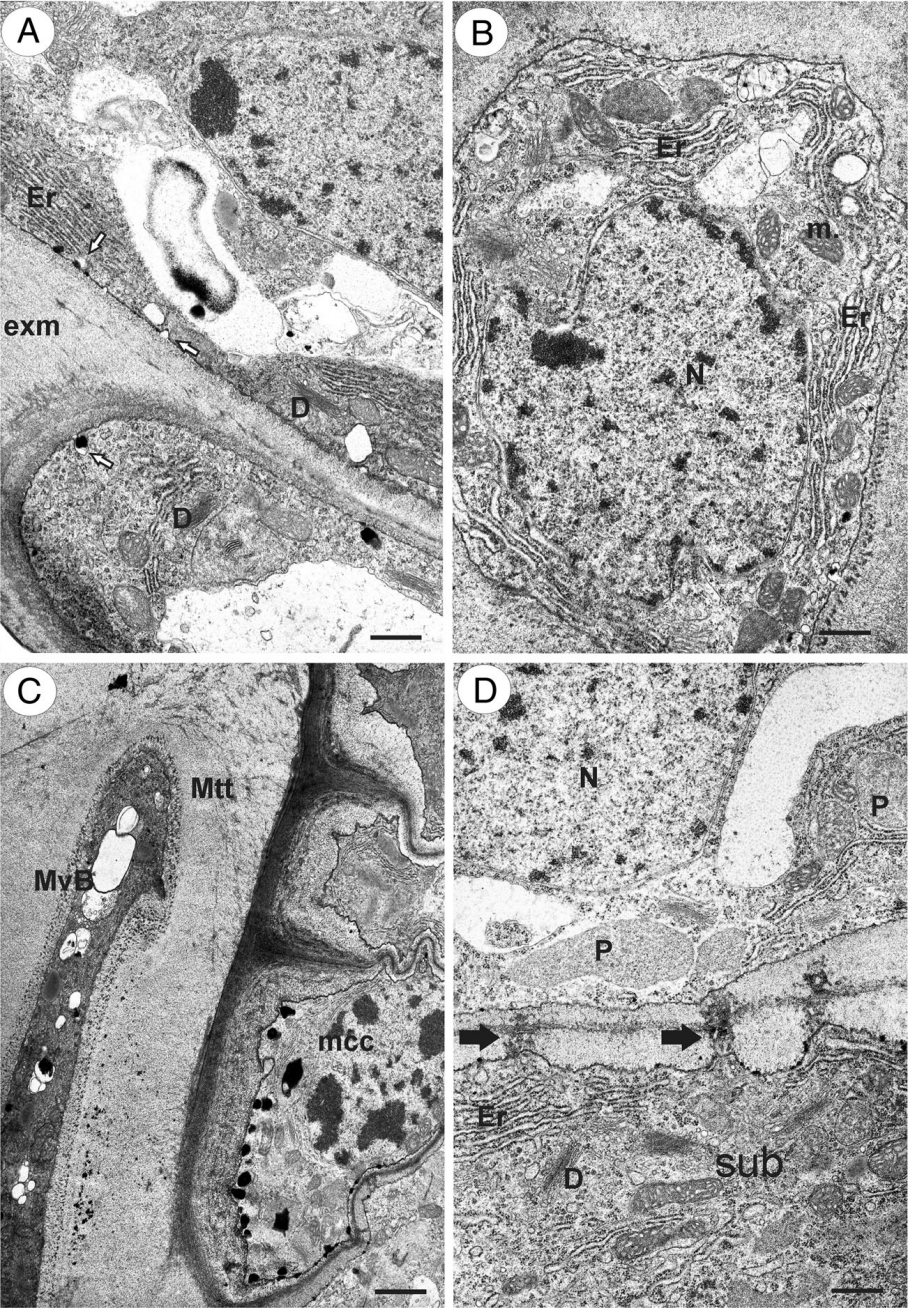
Ultrastructure of transmitting tissue in *T. tenuifolium.* **a** Exocytose of small vesicles (*white arrows*) in the transmitting tissue cells; ER cisternae (*Er*), dictyosomes (*D*), and extracellular matrix (*exm*). *Bar* = 0.6 μm. **b** Transverse section through an micropylar transmitting tissue cell; ER cisternae (*Er*), mitochondrion (*m*), nucleus (*N*), *bar* = 0.6 μm. **c** A part of a section through a micropyle near synergids; multivesicular bodies (*MvB*), micropylar transmitting tissue cell (*Mtt*), micropylar canal cell (*mcc*), *bar* = 1 μm. **d** A part of a section through subepidermal (*sub*) and micropylar transmitting tissue cells; plasmodesmata (*black arrow*), dictyosomes (*D*), ER cisternae (*Er*), nucleus (*N*), plastid (*P*), bar = 0.6 μm

The micropyle was asymmetrical. The part of the integument that was on the side that was proximal to the funicle had a differently developed epidermis than the one on the side that was distal to the funicle. The epidermis of the part that was adjacent to the micropyle on the funicle side formed the transmitting tissue (Fig. 1a, b), except at the level that was closest to the embryo sac (Fig. 1b). The micropylar canal was completely filled with the ovule transmitting tissue (obturator) and the matrix that was produced by these cells (Fig. 1c, d), and therefore, the micropyle was closed. The cells of the ovule transmitting tissue were elongated in the synergid direction (Fig. 1b). The transmitting tissue cells and their matrix reached to the apex of the synergids (Fig. 1b). There was a well-developed rough endoplasmic reticulum in the cytoplasm, which formed parallel stacks. Mitochondria were frequent and had well-developed cristae. Dictyosomes were also frequent (Fig. 2a, b). The exocytosis of small vesicles was observed (Fig. 2a). Plastids had electron-dense stroma and weakly developed internal membranes. The nucleus had an elongated shape. Myelin bodies, spherosomes, lipid bodies, and multivesicular bodies, which primarily occurred in the micropylar transmitting tissue cells that were near synergids, also occurred in the cytoplasm (Fig. 2c). There were branched plasmodesmata between the subepidermal cells and ovule transmitting tissue cells (Fig. 2d). Like the micropylar transmitting tissue cells, the subepidermal cells had a well-developed rough endoplasmic reticulum (Fig. 2d). The extracellular matrix had a positive reaction for insoluble polysaccharides (positive PAS reaction).

**Fig. 3:**
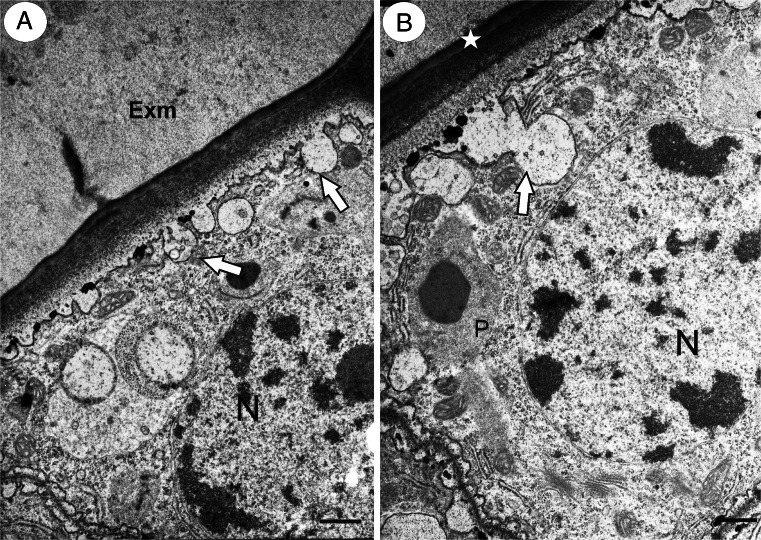
**a, b** Ultrastructure of micropylar canal cells in *T. tenuifolium*. Note the extracellular matrix (*Exm*) of micropylar transmitting tissue cells, cuticle (*star*), exocytose of small vesicles (*white arrows*), nucleus (*N*), and plastid (*P*). *Bar* = 0.75 μm

The epidermal cells of the integument part on the side that was distal to the funicle had a rectangular shape in that section. In contrast to the transmitting tissue cells, they were in close contact. There was no intercellular space that was filled with the extracellular matrix (Fig. 2c). The cuticle layer occurred between these cells and the micropylar transmitting tissue cells (Fig. 3a, b). The exocytosis of various vesicles that contained electron-dense material or smaller vesicles was also observed (Fig. 3a, b).

### Micropyle ultrastructure in apomictic species

**Fig. 4:**
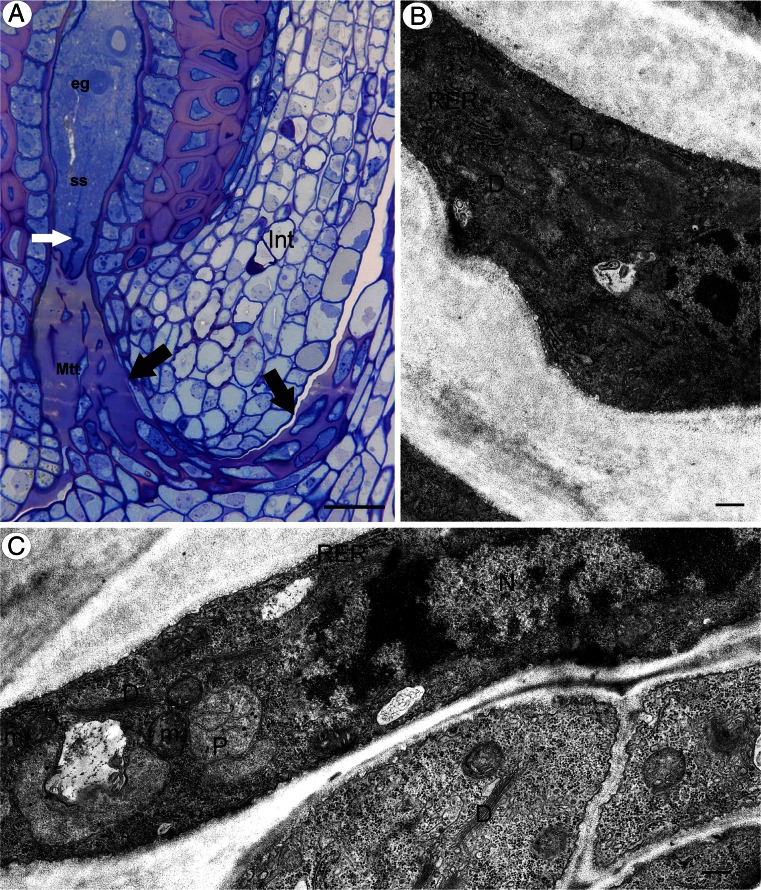
Transmitting tissue structure in apomictic dandelion *T. officinale s.l.* (clone SA-B). **a** Semithin section through an ovule and a part of an ovary showing the transmitting tissue (*Mtt*, *black arrow*), egg cell (*eg*), synergids (*ss*), and filiform apparatus (*white arrow*). *Bar* = 20 μm. **b, c** Ultrastructure of micropylar transmitting tissue cells, dictyosomes (*D*), ER cisternae (*RER*), plastid (*P*), and mitochondrion (*m*). *Bar* = 0.75 and 0.5 μm

The micropyle had a similar structure to that in amphimictic dandelions. The micropylar canal was completely filled with the micropylar transmitting tissue and the matrix that was produced by these cells (Fig. 4a, b). Transmitting tissue cells and their matrix reached to the apex of the synergids (Fig. 4a), which had a filiform apparatus. Like amphimictic dandelions, the micropylar transmitting tissue cells seemed to be active and had well-developed mitochondria, rough endoplasmic reticulum, and numerous dictyosomes (Fig. 4b, c). Plastids occurred but they had weakly developed internal membranes. There were also multivesicular bodies. The extracellular matrix had a positive reaction for insoluble polysaccharides (positive PAS reaction).

**Fig. 5:**
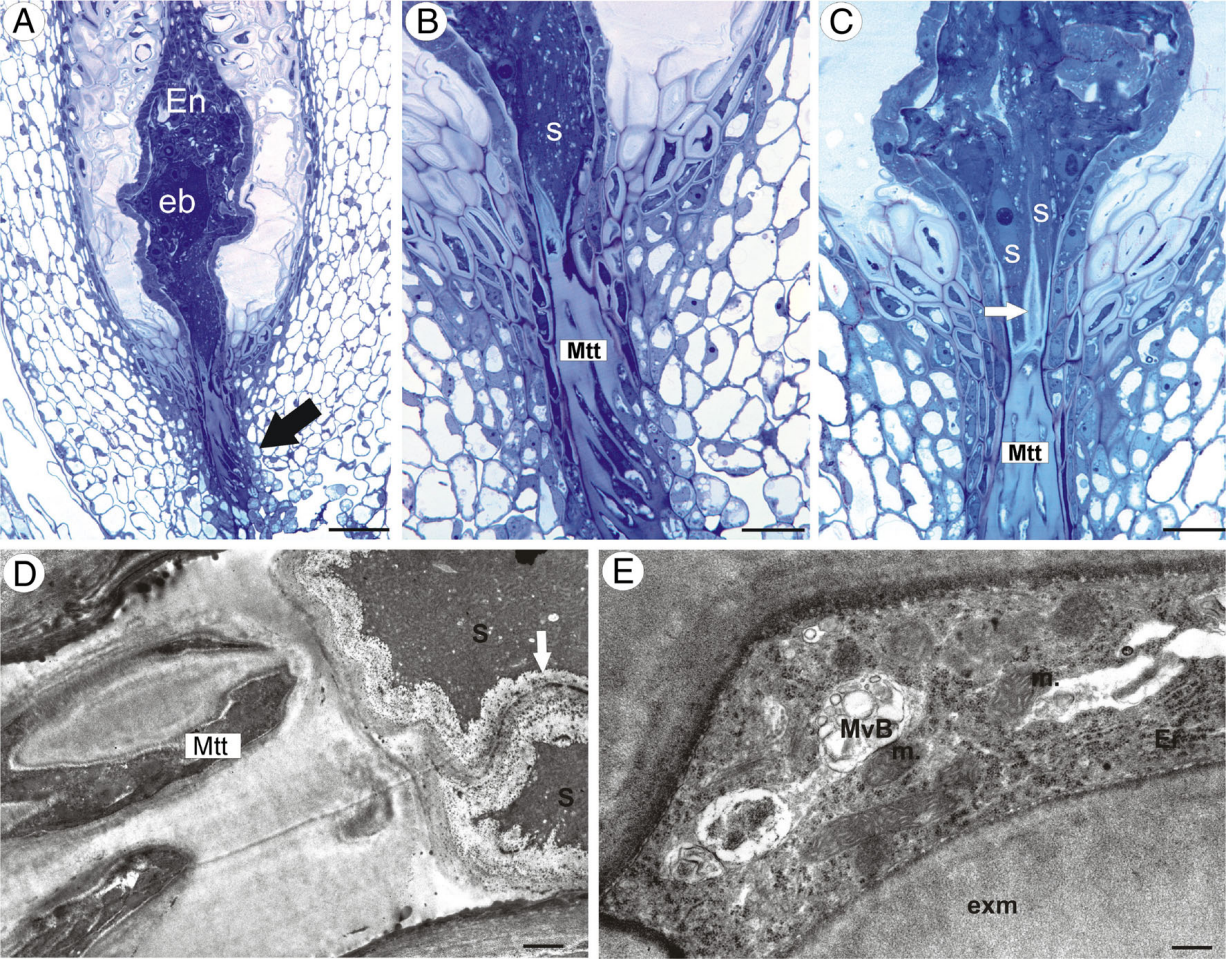
**a−c** Semithin section through a young seed of an apomictic dandelion *T. officinale* s.l. (clone Kraków-Podgórze) showing the micropylar transmitting tissue (*Mtt*, *black arrow*), cellular endosperm (*En*), globular embryo (*eb*), synergids (*s*), and filiform apparatus (*white arrow*). **a** *Bar* = 50 μm. **b, c** *Bar* = 20 μm. **d** Ultrastructure of micropylar transmitting tissue cells, note contact of micropylar transmitting tissue cells (*Mtt*) with synergids (*s*), filiform apparatus (*white arrow*), ER cisternae (*ER*), multivesicular bodies (*MvB*), extracellular matrix (*exm*), and mitochondrion (*m*). *Bar* = 1.8 and 0.4 μm

Developed embryos and endosperm tissue were observed in the ovules that were taken from the flowers of apomicts during anthesis (Fig. 5a). The transmitting tissue cells still had contact with synergids (Fig. 5b, c), which persisted for a long time without any degeneration in spite of the presence of an embryo and endosperm. Transmitting tissue cells were still alive in this stage with an ultrastructure that was similar to the one that was observed in the sexual species.

## Discussion

The general structure of the pollen transmitting tissue in *Taraxacum* is similar to that of other members of the Asteraceae family, e.g., from the genera: *Arnaldoa*, *Buphthalmum*, *Cichorium*, and *Helianthus* (Yan et al. 1991; Erbar and Leins 2000; Erbar and Enghofer 2001; Erbar 2003; Gotelli et al. 2010). The ovules in *Taraxacum* are more similar to the ovules of *C. intybus* and *B. salicifolium*, which have a shorter funiculus, than to *Arnaldoa* (Erbar 2003). Moreover, a *Helianthus* ovule has a very short funicle (Yan et al. 1991). However, as was mentioned above, detailed data about the ultrastructure of ovular transmitting tissue in Asteraceae are quite scarce.

Kościńska-Pająk (2006) suggested that the *Chondrilla* obturator cells lyses thus forming the extracellular matrix substance that filled the micropylar canal. However, in neither *Helianthus* (Yan et al. 1991) nor *Taraxacum* was the lyses of the micropylar transmitting tissue cells observed. An extracellular matrix substance was actively produced by the ovule transmitting tissue cells. Micropylar transmitting tissue cells still persisted in *Taraxacum*, despite the presence of an embryo (see also Fig. 4 in Płachno et al. 2014a). The micropylar transmitting tissue cell ultrastructure in *Taraxacum* is similar to that described for *Helianthus* (Yan et al. 1991) as well as for species from families other than Asteraceae (Tilton and Horner 1980; Tilton et al. 1984; Vardar et al. 2012). We did not find any major differences between the micropyle structures in members of different *Taraxacum* sections (*Palustria* versus *Taraxacum*).

The ultrastructure of the transmitting tissue has been studied in many species of angiosperms. Most of these studies have only been concerned with the stylar part (e.g., Gawlik 1984; Hu and Zhu 1990; Sage et al. 2009). However, studies on the ovule transmitting tissue have been limited to a very few taxa, e.g., *Helianthus* (Yan et al. 1991), *Lilium* (Singh and Walles 1995), and *Gagea* (Vardar et al. 2012). The stylar and ovarian transmitting tissues are anatomically and histochemically similar to the stigma. Our present observations confirmed that the micropylar canal is filled by ovule transmitting tissue and its extracellular matrix in both sexual and apomictic dandelions. The occurrence of an extracellular matrix was also described in another species from Asteraceae family, *H. annuus*, (Yan et al. 1991), and other angiosperms (e.g., Hristova et al. 2005; Sage et al. 2009 and literature cited therein). Some extracellular matrix molecules contain calcium, pectins, lipids, arabinogalactans/arabinogalactan proteins (AGs/AGPs), as well as other proteins, e.g., cysteine-rich adhesion (SCA), a lipid-like transfer protein (Mollet et al. 2000; Park and Lord 2003; Wu et al. 2000; Khosravi et al. 2003). According to our cytochemical results, the extracellular matrix consists of polysaccharides. Similar observations have been reported in the sunflower (Yan et al. 1991). Our present observations confirmed that the micropylar transmitting tissue cells in dandelions are rich in mitochondria, profiles of endoplasmic reticulum, plastids, and dictyosomes, multivesicular bodies, and lipid bodies, and these agree with the observation that was made in the sunflower (Yan et al. 1991). In addition, Vardar et al. (2012) showed that the transmitting tissue cells were rich in RER, dictyosomes, ribosomes, plastids, and mitochondria in *Gagea*. According to Raghavan (1997), cells with abundant ribosomes, mitochondria, endoplasmic reticulum, dictyosomes, and plastids are very active metabolically. Therefore, the ovule transmitting tissue is still active in apomictic dandelions despite the presence of an embryo and endosperm.

What are the evolutionary benefits of the presence of micropylar transmitting tissue cells in the ovules of autonomous apomictic dandelions (despite the fact that both endosperm and embryo development is independent of fertilization)?

One answer might be none. The hazy evolutionary history of the genus *Taraxacum* does not allow for the dating of the emergence of apomixis in the genus. Nevertheless, the majority of apomictic dandelions are considered to be young evolutionarily (Richards 1973). Richards (1973) suggested that the first apomictic dandelion species may have emerged early in the evolutionary history of the genus, although the main expansion of apomictic species is undoubtedly connected with the glacial ages, especially with the Pleistocene period (Richards 1973). From an evolutionary point of view, this may be a short time span for the development of strong architectural differences between the sexuals and apomicts. Thus, apomictic species have not had enough time to develop an evolutionary trend to reduce the “unnecessary costs” that are associated with sexual reproduction. For example, the majority of apomictic dandelions produce pollen, although it has no function for seed production, or the presence of synergids that have a similar ultrastructure and that have a filiform apparatus in both sexual and apomictic *Taraxacum* species (Płachno et al. 2014a). Recent research in another well-documented apomictic species group *Ranunculus auricomus* agg. in the genus *Ranunculus* placed the date of the divergence of the apomictic lineages of the *R* *anunculus carpaticola* × *cussubicifolius* from its sexual progenitor approximately 80 kyr ago (Pellino et al. 2013), while its sexual progenitors (*Ranunculus cassubicifolius* and *R. carpaticola*) diverged ca 317 kyr ago (Hörandl 2004). The transcriptomic data from the RNAseq showed that apomictic lineages were under divergent selection after the divergence from the sexual lineages. A comparison of the ratio of synonymous versus nonsynonymous mutations between sexual and apomictic species only identified some outlying loci in the apomictic lineages that were under divergent selection. These loci are mainly connected with the cell cycle and reproduction (Pellino et al. 2013). This suggests that divergent selection may favor some genes during the evolution process; however, it is questionable whether a selection may be strong enough to rebuild the ovule structure in apomictic species.

The second answer might be different—there are some evolutionary benefits of retaining the functionality of all of the necessary structures that are connected with pollination and fertilization for apomicts. This makes sense in the light of a possible hybridization among sexuals and apomicts and the formation of new apomictic clones (van Dijk 2003). Gene flow among apomictic and sexual dandelions has been documented repeatedly not only in the case in which apomicts represent pollen donors for the sexuals (Menken et al. 1995; van Dijk et al. 1999; van der Hulst et al. 2003; Mártonfiová 2006; Mártonfiová et al. 2007) but also vice versa. Fertilization of an unreduced egg cell (origin of B_III_ hybrids) in apomictic dandelions has been observed and documented repeatedly in *Taraxacum* (Małecka 1973; van Baarlen et al. 2002; Mártonfiová 2006). This means that gene flow can be bidirectional. Although fertilization of an unreduced egg cell of apomicts is a rather rare event, it may be an important process in generating new apomictic lineages that have higher ploidy levels. Such a process can be seen as one of the important processes of diversification within apomictic genera (e.g., Kirschner and Štěpánek 1996). It is questionable whether this is due to fact that the benefits are connected with gene flow, which are larger than the energy benefits that are connected with the reduction of unnecessary costs, or whether this is due to the young evolutionary age of apomictic dandelions. Apomictic dandelions did not reduce some of the structural and developmental characters that are not effectively used by apomictic individuals. Our results show that even the ovule of apomicts that has an endosperm and embryo has vital transmitting tissue cells; thus, there is no barrier for the pollen tube to reach the ovule. The main difference is in the timing of the embryo development. While in apomicts the majority of embryos are already developed before the anthesis (van Baarlen et al. 2002), in sexual species, embryos develop only after fertilization.

In conclusion, we think that this is the first time in the literature that the micropyle ultrastructure in sexual and apomictic taxa from one genus is presented. The results of our comparative observations did not reveal any differences in the micropyle anatomy and ultrastructure of the ovules between sexual and apomictic species within the genus *Taraxacum*. However, the presence of vital micropylar transmitting tissue cells makes it possible for an unreduced egg cell of apomicts to be fertilized. Such a process may be an important in generating new apomictic lineages.
